# Molecular Evolution and Functional Analysis of Rubredoxin-Like Proteins in Plants

**DOI:** 10.1155/2019/2932585

**Published:** 2019-07-02

**Authors:** Ying Li, Pan pan Liu, Xin Ni

**Affiliations:** Key Laboratory of Saline-Alkali Vegetation Ecology Restoration in Oil Field (SAVER), Ministry of Education, Alkali Soil Natural Environmental Science Center (ASNESC), Northeast Forestry University, Harbin, China

## Abstract

Rubredoxins are a class of iron-containing proteins that play an important role in the reduction of superoxide in some anaerobic bacteria and also act as electron carriers in many biochemical processes. Unlike the more widely studied about rubredoxin proteins in anaerobic bacteria, very few researches about the function of rubredoxins have been proceeded in plants. Previous studies indicated that rubredoxins in* A. thaliana* may play a critical role in responding to oxidative stress. In order to identify more rubredoxins in plants that maybe have similar functions as the rubredoxin-like protein of* A. thaliana*, we identified and analyzed plant rubredoxin proteins using bioinformatics-based methods. Totally, 66 candidate rubredoxin proteins were identified based on public databases, exhibiting lengths of 187–360 amino acids with molecular weights of 19.856–37.117 kDa. The results of subcellular localization showed that these candidate rubredoxins were localized to the chloroplast, which might be consistent with the fact that rubredoxins were predominantly expressed in leaves. Analyses of conserved motifs indicated that these candidate rubredoxins contained rubredoxin and PDZ domains. The expression patterns of rubredoxins in glycophyte and halophytic plant under salt/drought stress revealed that rubredoxin is one of the important stress response proteins. Finally, the coexpression network of rubredoxin in* Arabidopsis thaliana* under abiotic was extracted from ATTED-II to explore the function and regulation relationship of rubredoxin in* Arabidopsis thaliana*. Our results showed that putative rubredoxin proteins containing PDZ and rubredoxin domains, localized to the chloroplast, may act with other proteins in chloroplast to responses to abiotic stress in higher plants. These findings might provide value inference to promote the development of plant tolerance to some abiotic stresses and other economically important crops.

## 1. Introduction

Rubredoxin, a nonheme iron protein first discovered and isolated from* Clostridium pasteurianum*, is one of the most simple iron-sulfur (Fe-S) proteins [1, 2]. Previous studies have shown that rubredoxin contains a single Fe, no sulfur, and one iron atom which is coordinated by four conserved cysteine residues [3, 4]. As that inorganic sulfide is not included, there is a sharp contrast between rubredoxin and other members of the nonheme iron protein [Fe(SCys)4] family. In some anaerobic bacteria, rubredoxin plays an important role in the reduction of superoxide [5–8] and has also been shown to act as electron carriers in many biochemical processes (including the assembly of photosystem II), carbon fixation, fatty acid beta-oxidation using acyl-CoA dehydrogenase, and lipid homeostasis [4, 5, 9, 10]. It is indicated that rubredoxin could protect* Pyrococcus furiosus* and* Desulfovibrio vulgaris* from oxidative stress by functioning as an electron donor to superoxide reductase reaction system (SOR) [11–13]. Unlike the more widely studied about rubredoxin proteins in anaerobic bacteria, very few researches about the function of rubredoxins have been proceeded in plants. As recorded in Interpro up to 2019/05/10, there are 311 proteins carrying the rubredoxin domain in Viridiplantae catalogue (http://www.ebi.ac.uk/interpro/entry/IPR024935/taxonomy).

Previous studies indicated that rubredoxin proteins in plant might respond to adversity. In* Arabidopsis thaliana*, a rubredoxin-like protein encoded by* ENH1* and localized to the chloroplast was shown to increase sensitivity to oxidative stress [14]. Furthermore, our recent study indicates that a rubredoxin-like protein from* Puccinellia tenuiflora* (PutRub) may increase the salt tolerance by reducing the accumulation of ROS [15, 16]. It was reported that the overexpression of the enhancer of SOS3-1 from tobacco and the salt tolerant variety CS52 of* Brassica juncea* could protect plants from salt stress by excluding Na^+^ from the cytosol to reestablish ion homeostasis [14, 17]. Thus, it is reasonable to infer that rubredoxin might play a key role in plant adaptability to adversity environmental stress. As the critical role for rubredoxin in plant response to adversity, identifying more candidate rubredoxin proteins involved in plant tolerance and resistance to abiotic stresses may proceed to promote the researches about improving plant tolerance to abiotic stresses. Therefore, in the present study, we identified rubredoxin in plants using bioinformatics-based methods to explore the genetic characteristics and potential roles of rubredoxin in higher plants.

In conclusion, we identified 66 candidate rubredoxin proteins in plants that have similar sequence structures and functions to the rubredoxin-like protein of* Arabidopsis thaliana* with BLASTP. By applying bioinformatics-based methods and software, we detected their general chemical-physical and genetic characteristics. Additionally, we also analyzed the mRNA expression patterns of the identified rubredoxin-like proteins in response to abiotic stress in* Arabidopsis thaliana*,* Arabidopsis lyrata*,* Oryza sativa*,* Zea mays*, and* Eutrema salsugineum*. The result of At5g17170 coexpressed network indicated that several coexpressed genes that worked in conjunction with rubredoxin in the salt stress response. According to the aforementioned description, our study may not only lay the foundation for further functional studies of rubredoxin in plant species, but also provide a value reference that rubredoxin might be a promising and untapped genetic resource for plant improvement and could be deployed further in the development of plant tolerance to some abiotic stresses and other economically important crops.

## 2. Materials and Method

### 2.1. Retrieval of Rubredoxin-Like Family Proteins from Existing Databases

Rubredoxin family protein (NP_568342.1) from* Arabidopsis thaliana* was set as the query sequence to search against the nonredundant protein sequence database in NCBI (nr database) [18] in running BLASTP (2.3.0) (E-value = 1e-5, identity = 50%). A pairwise alignment of these subject sequences from the result of BLASTP was carried out to remove redundancy (E-value = 1e−5, identity ≥ 98%). GRAVY (grand average of hydropathicity scores) (http://www.expasy.org/tools/protparam.html) were used to predict the grand average of hydropathicity scores, PFAM (http://pfam.xfam.org/) [19], and the Conserved Domain Database (https://www.ncbi.nlm.nih.gov/Structure/cdd/wrpsb.cgi/) [20] were used to validate the presence of the PDZ and rubredoxin-like domains.

### 2.2. Sequence and Motif Analyses of Rubredoxin-Like Proteins

ProtParam (http://web.expasy.org/protparam/) [21] was used to calculate the general physical and chemical parameters of the retrieved rubredoxin-like candidate sequences. Four subcellular location predict tools, TargetP 1.1 (http://www.cbs.dtu.dk/services/TargetP/), WolfPSORT (http://www.genscript.com/wolf-psort.html), Multi-Schlo, and BUSCA were applied to predict the subcellular location of the candidate proteins in this study [22–25]. MEME v4.11.2 (http://meme-suite.org) [26] was used to identify conserved motifs in the putative proteins using the following parameters: 0-order model of sequences, maximum number of motifs = 5, and optimum motif width constrained between 6 and 100 residues.

### 2.3. Multiple Sequence Alignments and Phylogenetic Tree Construction

Alignments of protein sequences were created in MEGA 7.0 after running ClustalW [27] with the following parameters: gap opening penalty = 10 and gap extension penalty = 0.2 [10]. A phylogenetic tree was constructed by MEGA 7.0 using the neighbor-joining (NJ) method with 1000 bootstrap replicates.

### 2.4. Expression Analyses of Rubredoxin in Higher Plants

Expression data for* A. thaliana* and* Oryza sativa* were downloaded from the GEO database (https://www.ncbi.nlm.nih.gov/geo/) to explore the expression patterns of rubredoxin in different tissues and over the course of development. Here, the expression characteristics of rubredoxin in* A. thaliana*,* A. lyrata*,* O. sativa*, and* Zea mays* [28] were analyzed in the face of various abiotic stressors. The expression array of* Eutrema salsugineum* under salt stress was retrieved from GEO [29]. The accession numbers and sample information of gene expression arrays are listed in Table S1.

### 2.5. Coexpression Network of Rubredoxin in A. thaliana under Abiotic Stress

In order to explore the coordinated regulation of genes with rubredoxin in* A. thaliana* (http://atted.jp/) [30] coexpression data were extracted from ATTED-II. At5g17170, encoding NP_568342.1, was used as a query to search ATTED-II under abiotic conditions (MR < 50), and the genes that passed the threshold were used as queries to extract coexpressed genes in ATTED-II under abiotic conditions (MR < 20). Then the coexpression network of At5g17170 was constructed in Cytoscape 3.4.0 [31]. The functions of genes in the coexpression network were annotated using DAVID (https://david.ncifcrf.gov/) [32].

## 3. Results

### 3.1. Retrieval and Analysis of Rubredoxin-Like Protein Sequences

The use of NP_568342.1 as query to search against the nr database in NCBI via a local BLASTP yielded 96 putative rubredoxin-like proteins. Combined with the conserved domains predicted by PFAM and the CD search, 64 candidate proteins with rubredoxin and PDZ domains were selected for further analyses. In addition to the 64 candidate proteins identified from public databases, there are two public database unaccession proteins that were included in the study: rubredoxin-like family proteins from* Puccinellia tenuiflora* (PutRub) and* Salix mongolica* (SaRub) isolated in our laboratory. PutRub was selected from the cDNA library of* Puccinellia tenuiflora* under NaHCO_3_ stress and SaRub was identified from the transcriptome of* Salix mongolica* by using a homology search. Information such as protein accession numbers, gene, locus_tag, gene length, exon number, and genomic positions is listed in Table S2.

The length of these candidates ranged from 187 to 360 amino acids with molecular weights of 19.856–37.117 kDa. The majority of candidate proteins had a pI value greater than 9. The GRAVY scores of all candidate rubredoxin were negative, implying that these proteins were all hydrophilic proteins. The majority of candidate proteins were predicted be localized to the chloroplast. The information on the physical and chemical properties and predicted domains of all rubredoxin-like proteins are listed in Table S3.

### 3.2. Conserved Motif Analyses of Rubredoxin-Like Proteins

**Figure 1:**
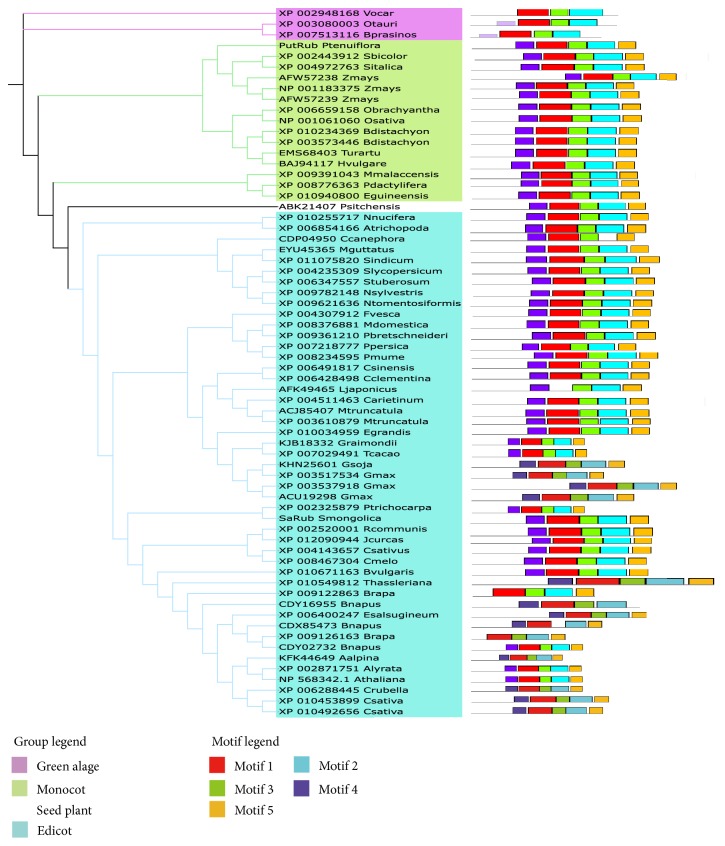
Distribution of predicted motifs in plant rubredoxins.

Further analyses show five conserved motifs of plant rubredoxins in the 66 plants (Table 1). In this analysis, we observed that most rubredoxins exhibited similar motif-patterns between species, with the motif order as follows: 4-1-3-2-5 (Figure 1). Motif 1 was composed of 49 amino acids, motif 2 was 53 amino acids long, and motifs 3 and 4 had 29 amino acid residues. The combined results of MEME and PFAM showed that detected motifs 2 and 4 matched well with the rubredoxin (rubredoxin-like) domain and the PDZ/PDZ-signaling domain, respectively. Motif 2 contained four conserved cysteine residues, similar to rubredoxin domain, as it is known that the iron atom in plant rubredoxin is coordinated by four conserved cysteine residues [33, 34]. Motif 4 was present in all 66 candidate proteins and exhibited a high degree of similarity to PDZ or the PDZ-signaling domain in the PDZ superfamily of proteins. It is also interesting to note that motif 5 was not found to be present in rubredoxin from green algae.

### 3.3. Multiple Sequence Alignment and Phylogenetic Analysis of Rubredoxin-Like Proteins

**Figure 2:**
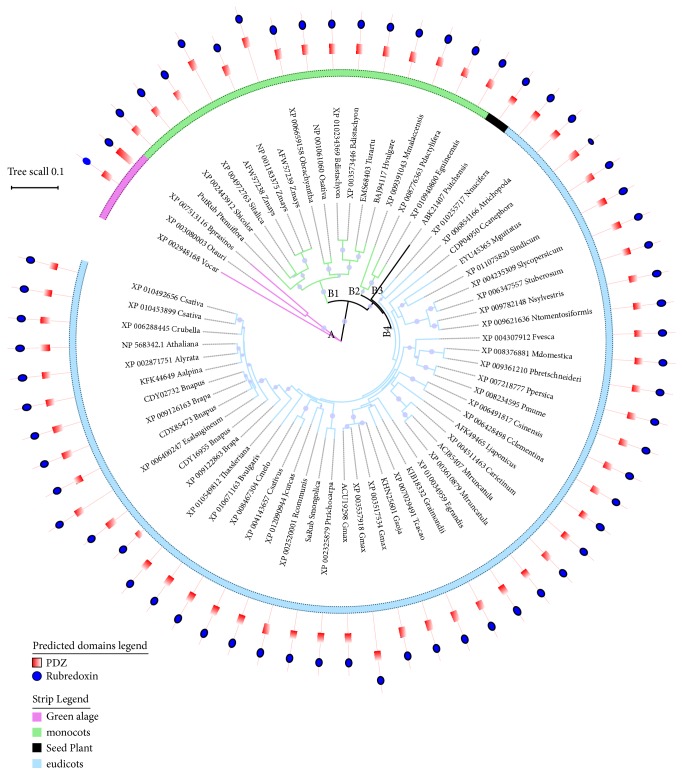
Phylogenetic analysis of rubredoxin-like proteins.

To investigate the relationship of rubredoxin proteins between various species of higher and lower plants, a neighbor-join tree was constructed using MEGA 7.0 and included 66 candidate proteins from 56 organisms. The constructed tree was divided into two main groupings (groups A and B), where group A contained only three sequences from lower plants (green algae) and group B contained monocots, seed plants, and eudicots. Group B was further subdivided into three subgroups: B_1_, B_2_, and B_3_ based on subclustering patterns within the tree. Previous research has demonstrated that plant rubredoxins were historically derived from the primary endosymbiosis of a cyanobacterium [4], and according to the evolutionary tree in the present study, rubredoxin family proteins in higher plants may have evolved from green algae. In subgroups B1 and B2, rubredoxin from monocots and eudicots clustered together. We also found that rubredoxin from closed evolutionary species exhibited higher bootstrap support values (Figure 2). This figure shows a phylogenetic analysis, constructed using the neighbor-joining method (1000 bootstrap replicates), resulting from an alignment of amino acid sequences of 66 putative rubredoxin-like proteins. Circle size is proportional to bootstrap values, while branch color indicates different species. Strips within each circle indicate the species, where each species is assigned a specific color.

### 3.4. Tissue-Specific Expression of Rubredoxin over the Course of Development

**Figure 3:**
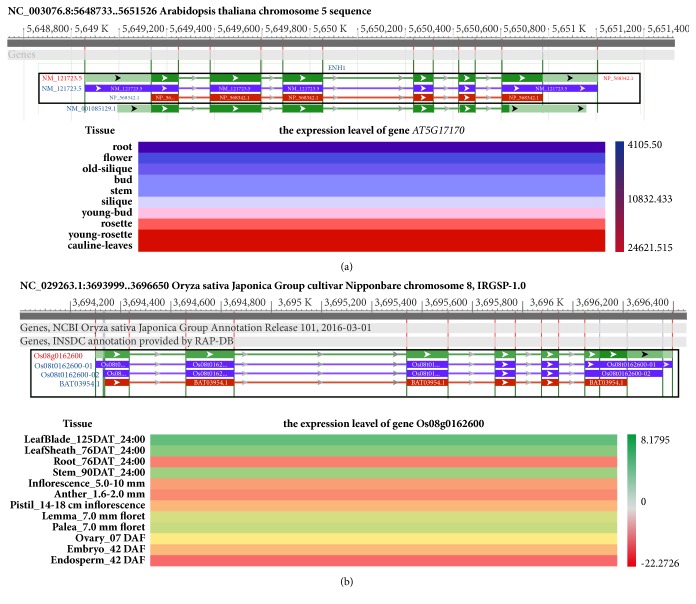
*Expression patterns of genes encoding rubredoxin in Arabidopsis thaliana and Oryza sativa*. (a) UCSC genome browser and expression patterns of At5g17170 in diverse organs and during development in* A. thaliana*. (b) Genome browser and expression patterns of Os08g0162600 in different tissues, during development in* O. sativa*.

To gain insight into the possible function of rubredoxin genes during development, we chose two model plants* A. thaliana* and* O. sativa* to explore tissue-specific expression patterns of genes encoding rubredoxin during different development stages. Datasets GSE34188 for* A. thaliana* and GSE21494 for* O. sativa* were chosen to analyze the expression patterns of genes encoding rubredoxin, and two heat maps were subsequently created using GenePattern (http://software.broadinstitute.org/cancer/software/genepattern/) [35] for different developmental stages and tissues (Figure 3). In* A. thaliana*, the expression of* At5g17170*, which encodes rubredoxin family protein, NP_568342.1, was lower during the development of buds, siliques, and rosettes than in young buds, siliques, and rosettes. This indicates that* At5g17170* may play a role in the development of tissues (Figure 3(a)). A similar analysis for* Os08g0162600* (Figure 3(b)), resulting in expression patterns similar to those observed in the leaves of* A. thaliana*, showed that Os08g0162600 was also weakly expressed in the root and endosperm and more strongly expressed in leaves. In summary, although we found that* At5g17170* and* Os08g0162600* were expressed in most tissues (i.e., root, stem, and leaf),* At5g17170* and* Os08g0162600* exhibited the highest expression in leaves and the lowest levels of expression in root tissues. The predominant expression of rubredoxin in leaves is consistent with its subcellular location to the chloroplast and suggests that rubredoxin may play a role in the functions of the chloroplast.

### 3.5. Expression Analysis of Genes Encoding Rubredoxins under Conditions of Abiotic Stress in Typical Plants

To explore the potential functions of rubredoxin in response to abiotic stress in glycophytic plants,* A. thaliana* and* A. lyrata* from the eudicots and* O. sativa* and* Z. mays* from the monocots were used to generate the expression profiles of rubredoxins in the face of various abiotic stressors.

**Figure 4:**
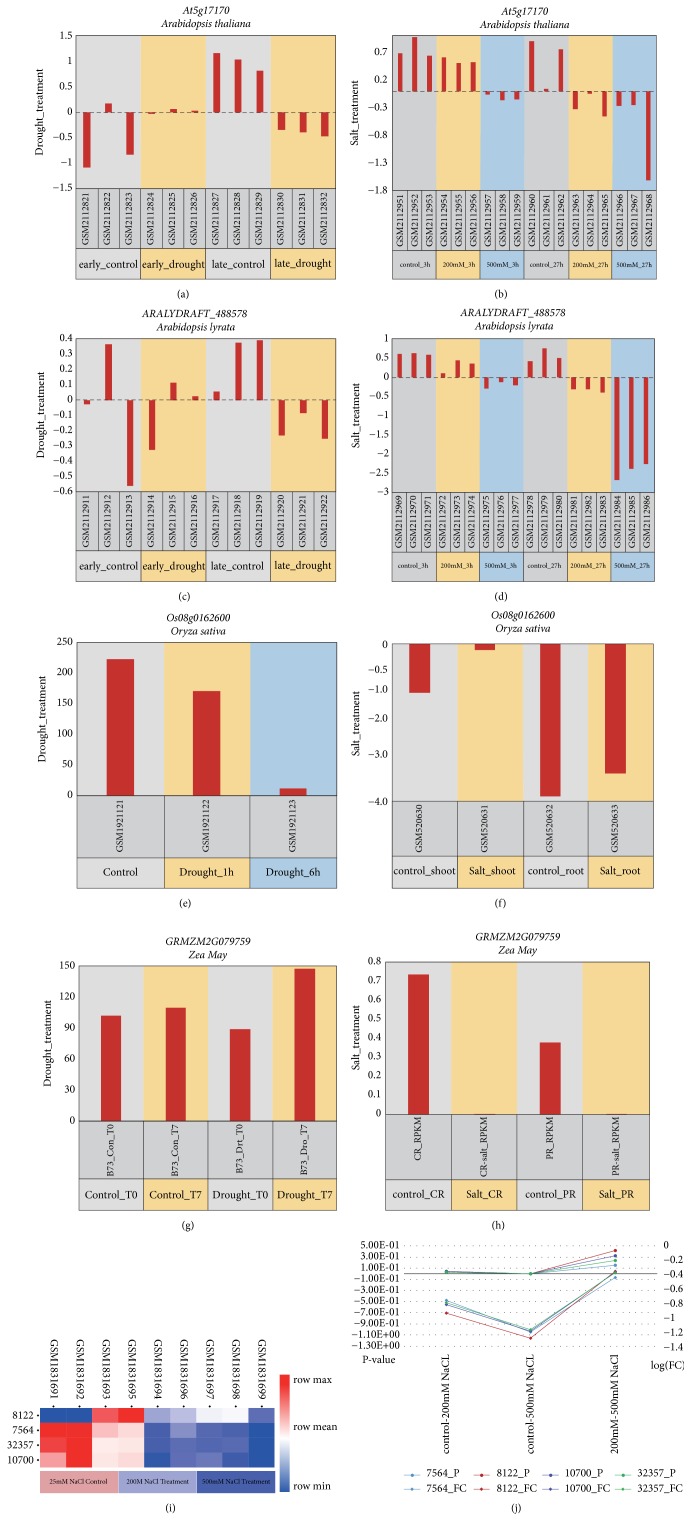
*Expression profile of genes encoding rubredoxin in plants under abiotic stress*. (a) and (b) indicate the expression profile of At5g17170 from* A. thaliana* under drought and salt stress, respectively. (c) and (d) show the expression profiles of ARALYDRAFT_488578 from* A. lyrata* under drought and salt stress. (e) and (f) show the expression patterns of Os08g0162600 from* O. sativa* under drought and salt stress. (g) and (h) show the abundance of GRMZM2G079759 in* Z. mays* under drought and salt stress. The horizontal axis indicates the accession number of samples in (a)–(h), while the vertical axis indicates the profile value of genes in corresponding samples with corresponding platforms from (a) to (h). (i) Heatmap of probes that are homologous to EUTSA_v10014045mg in E. salsugineum. (j) Statistics resulting from the DEG analysis with GEO2R for the corresponding probe. The horizontal axis indicates groups, the left vertical axis shows the p values for the DEG analysis, and the right vertical axis indicates the log (FC) of the DEG analysis.

The datasets GSE80099 and GSE80114 for* A. thaliana* were used to explore the expression of gene* At5g17170* (encoding NP_568342.1) under conditions of drought and salt stress, respectively. Under conditions of drought stress, we observed that the expression of* At5g17170* in shoots was not significantly different between the control and early-drought treatments (p value = 0.135) (Figure 4(a)). Meanwhile, we found that the expression of* At5g17170* in shoots was significantly lower in the late-drought group comparing to the control plants (p value = 1.63e-04, adjusted p value = 0.002). In analyzing GSE80114 for salt stress, we found that the expression of transcript* NM_121723* corresponding to NP_568342.1 did not exhibit significant changes in shoots after 3 h of treatment with 250 mM NaCl (Figure 4(b)). However, over longer periods of salt treatment, the expression of NM_121723 decreased significantly (p value = 2.89e−02, log (FC) = −0.83879692). On the other hand, when exposed to increasing concentrations of NaCl,* At5g17170* also showed significant changes when exposed to 500 mM for 3 h and 27 h in comparison to control plants. Interestingly, the same expression patterns were observed in* A. lyrata* for the PDZ and rubredoxin domains of* ARALYDRAFT_488578* (encoding chloroplast protein XP_002871751.1) under drought and salt stress, respectively (Figures 4(c) and 4(d)). The results of the expression patterns of genes encoding rubredoxin in* A. thaliana* and* A. lyrata* under conditions of abiotic stress indicate that these genes exhibited significant changes between control and treatment groups with both increasing time and the severity of stress, in agreement with previous observations [25]. This implies that rubredoxin could be involved in the biological processes that are engaged in response to salt and drought stress in eudicots.

In addition, the monocots* O. sativa* and* Z. mays* were used to analyze rubredoxin expression under conditions of abiotic stress. Datasets GSE74465 and GSE20746 were used to analyze* Os08g0162600* (encoding the rubredoxin protein) in rice subjected to drought and salt stress, respectively. We found that after 1 h of drought treatment, the whole plant expression of* Os08g0162600* did not change significantly (p value = 0.007342, log (FC) = 0.3927), while after 6 h, the expression of* Os08g0162600* significantly decreased in response to drought in comparison to control plants (p value = 2.2e−16, log⁡(FC) = −4.3265) (Figure 4(e)). In response to salinity, the expression of* Os08g0162600* significantly increased in shoots in comparison to the control group, while the expression of Os08g0162600 did not exhibit any significant changes in root tissues (Figure 4(f)). The significant increase in the expression of Os08g0162600 in shoot tissues in response to salt stress indicates that rubredoxin may also play a role in the salinity stress response in leaves, similarly to rubredoxin in* A. thaliana*. In summary, the results of the expression profile of Os08g0162600 in response to salt and drought stress demonstrate that rubredoxin may also be an important factor in salt and drought tolerance in rice. We used datasets GSE71046 and GSE53995 to analyze the gene the expression patterns of* GRMZM2G079759* (encoding NP_001183375.1) in* Z. mays* exposed to drought and salt stress. As a result, we observed that although the expression of* GRMZM2G079759* decreased in response to 10 d of drought conditions, this decrease was not significant in comparison to the control treatment. However, after 7 d of recovery, expression levels of GRMZM2G079759 significantly increased in the corn in comparison to plants that experienced 10 d of drought (Figure 4(g)). In addition, we observed that the expression of GRMZM2G079759 significantly decreased in crown and primary roots under conditions of salt stress (Figure 4(h)). Even though rubredoxin in maize did not exhibit significant changes when exposed to salt stress or drought, the overall levels of expression increased significantly in maize during recovery.

The aforementioned analyses indicate that rubredoxin may play an important role in the responses of glycophytic plants to abiotic stressors; it is also reasonable to infer that rubredoxins might also participate in pathways associated with environmental adversity in halophytes. Thus, we chose the typical halophilous plant* E. salsugineum* to further explore the expression patterns of rubredoxin under conditions of abiotic stress. Here, we analyzed the expression of the gene encoding XP_006400247.1 in* E. salsugineum*, using dataset GSE71271, under control (25 mM NaCl) and high salt conditions (250 mM NaCl and 500 mM NaCl). In this array, there were four probes homologous to* EUTSA_v10014045mg* (encoding XP_006400247.1) in* E. salsugineum*. We used GEO2R to identify whether these four probes are differentially expressed between the control and salt treatments group, and a visualized hierarchy cluster was constructed to display the expression patterns of EUTSA_v10014045mg (Figure 4(i)). The values of statistical analyses are shown in Figure 4(j). In Figures 4(i) and 4(j), we observed that the expression of the gene coding XP_00640024.1 decreased with increasing salt concentrations, where the changes in* EUTSA_v10014045mg* expression were not significant when in the 200 mM NaCl treatment and were significantly lower in the 500 mM NaCl treatment. Thus, according to these findings, rubredoxin may also participate in the response to salt stress in* E. salsugineum*. The results of our recent study using* P. tenuiflora*—a graminaceous and alkali-tolerant halophyte species—suggest that* P. tenuiflora* rubredoxin (PutRub) may play an important role in maintaining normal electron transfer to enhance the adaptability of transgenic plants to adversity and in the reduction of ROS accumulation under conditions of NaCl and NaHCO_3_ stress [16].

In summary, the analysis of expression in response to salt or drought stress indicates that rubredoxin did not change significantly in the early stages of salt or drought stress. However, as both the time of exposure and the treatment concentration increased, the expression of rubredoxin decreased significantly. From these results, it is reasonable to infer that rubredoxin may play a key role in the plant response to abiotic stress.

### 3.6. Coexpression of Gene Encoding Rubredoxin in A. thaliana

**Figure 5:**
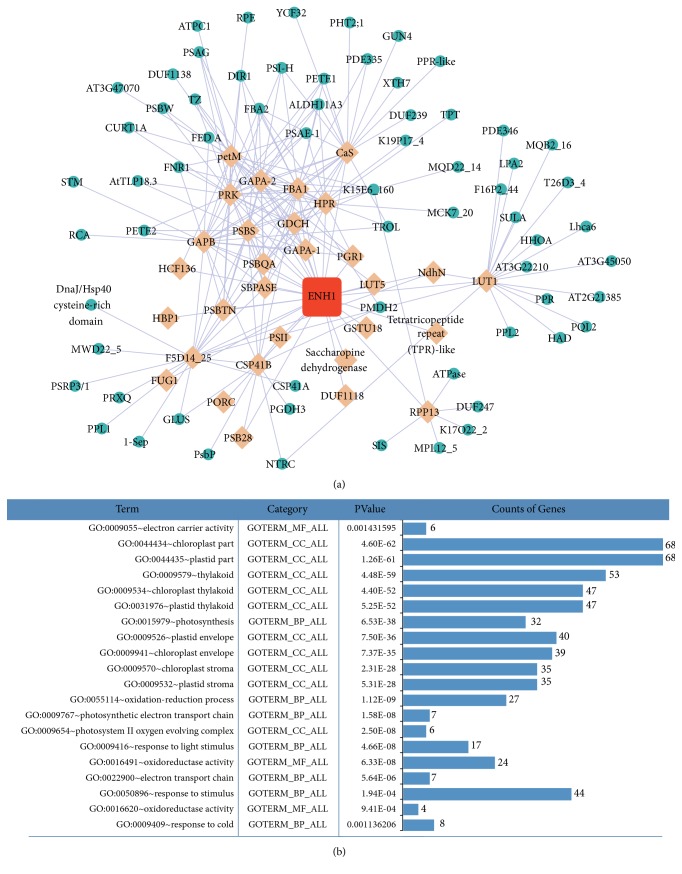
*Coexpression network of At5g17170 under abiotic stress and functional annotation using DAVID*. (a) This shows the coexpression network of At5g17170 under abiotic stress. Nodes in the network represent coexpressed genes and edges indicate significant levels of coexpression between any two given genes, where the width of edge is negative with respect to the MR value. Nodes with orange color indicate genes coexpressed with At5g17170, while green nodes represent genes coexpressed with genes are indicated by orange nodes. (b) The functional annotation table was created using DAVID.

The coexpression network of* At5g17170* (encoding rubredoxin) in* A. thaliana* was constructed to investigate gene-gene interactions and to elucidate the regulatory relationship of rubredoxin genes. First, we used* At5g17170* as a query to search ATTED-II, which yielded 30 genes coexpressed with* At5g17170 *under abiotic conditions (MR < 50). Subsequently, these genes were used as queries to search ATTED-II to obtain corresponding coexpressed genes (MR < 20). In the end,* At5g17170 *(ENH1), 30 genes coexpressed with* At5g17170* and 65 genes coexpressed with the 30 genes coexpressed with* At5g17170* were selected to construct a coexpression network in Cytoscape 3.4.0 (Figure 5(a)).

The coexpression network of* At5g17170* contained 96 nodes, representing genes, and 216 edges, which represent significant coexpression between any two given genes. These data were then uploaded to DAVID to explore their functional properties. As a result, we found that 16 of 30 genes coexpressed with At5g17170 were related to chloroplast function and the functioning of photosystem II, such as* CSP41b* (chloroplast stem-loop-binding protein of 41 kDa),* GAPB/A* (glyceraldehyde 3-phosphate dehydrogenase beta subunit),* HCF136* (photosystem II stability/assembly factor, chloroplast),* PRK* (phosphoribulokinase),* PSBQ* (extrinsic subunit of the photosystem II),* PSBS* (chlorophyll A-B binding family protein),* LHCb4.3* (extrinsic subunit of the photosystem II), and* SBPASE* (an isoform of light-harvesting complex). Three other genes play key roles in oxidoreductase activity, such as* PORC* (protochlorophyllide oxidoreductase C),* HPR* (hydroxypyruvate reductase), and* Ndhn* (an oxidoreductase that acts on NADH or NADPH, quinone, or a similar compound as acceptor). The results of the functional annotation also showed that two coexpressed genes with* At5g17170* are involved in electron carrying (*enh1*;* Cas*: calcium sensing receptor). The functions of the other six genes (*DUF1118, LUT1/5, PGR1, PSB28, and Saccharopine dehydrogenase*), coexpressed with* At5g17170* have not yet been analyzed under conditions of abiotic stress in plants. With the results of DAVID function annotation, we found that 68 of 96 genes were assigned into the term of chloroplast part (Figure 5(b)). In summary, 71% of the genes in the coexpression network were reported to be involved in biological process and signaling pathways that are involved in the response to abiotic stress. As the result of an enrichment analysis using DAVID, all of the top three clusters were related to chloroplast functions and photosystem II, which may play critical roles in maintaining chloroplast and thylakoid structural integrity to retain good photosynthetic capacity (Table S4).

## 4. Discussion

As it is well known that the sequence of a given protein is integral to its structure and function, the current study identified and filtered rubredoxin-like family proteins based on sequence similarity and conserved functional domains. In this study, the expression patterns of rubredoxin during different developmental stages and in different tissues showed that rubredoxin is predominantly expressed in leaves and is weakly expressed in other tissues. The results also show that majority of the identified sequences were localized to the chloroplasts and thylakoids, which is consistent with our findings that rubredoxin is most highly expressed in leaves and previous studies [15, 16, 25]. In eukaryotic cells, newly synthesized proteins can only function well in their appropriate subcellular locations [36]; therefore the subcellular location of a protein is a key feature to explore in the functional characterization of proteins the further exploration of protein-protein interactions in the cellular network system [37, 38]. In combination with the results of the subcellular localization and expression patterns of rubredoxin, it is likely that rubredoxin is involved in the function of chloroplast. Furthermore, the functional analysis of genes coexpressed with* At5g17170* showed that 16 of the coexpressed genes (*CSP41b, GAPB, GAPA, HCF136, PRK, PSBQ, PSBS, LHCb4.3,* and* SBPASE*) are also involved in the functioning of the chloroplast. These nine coexpressed genes with* At5g17170* were reported to participate in responses to at least one abiotic stress. Previous studies have indicated that* CSP41b* in* E. salsugineum* may be a putative target gene of miR399f in conditions of salt stress and ABA signaling which plays a key role in the maintenance of chloroplast functionality and confers heat and salinity stress tolerance in* A. thaliana* [39, 40]. These findings suggest that the maintenance of* CSP41b* expression could protect plants from injury owing to salinity and heat stress. In wheat, the expression of* GAPA* and* GAPB*—two types of glyceraldehyde-3-phosphate dehydrogenase—could be induced by at least one abiotic stress, where* GAPA/B* expression profiles have demonstrated that* GAPA/B* function beyond their key roles in glycolysis, most notably in abiotic stress resistance in plants [41]. In addition,* GAPB* in* Thellungiella halophila* functions to maintain photosynthetic efficiency and higher recycling rates of ADP and NADP (+) in order to decrease ROS production under saline conditions during plant development [42]. Studies have shown that* HCF136* has been implicated in photosynthetic redox signals that are involved in the control of ROS detoxification and play a key role in the assembly and repair of photosystem II [43–45]. A recent study about* A. thaliana* has also shown that* PSBS* and* PSBQ* take participate in preventing photo-damage to PSII under drought stress, in agreement with the results of the present study regarding the coexpression of* PSBS, PSBQ, LHCb4.3*, and* SBPASE*. [46]. The expression of* AcPsbQ1* from* Atriplex canescens* has been shown to change significantly in response to drought and salt stress, where the results of an expression profile for* AcPsbQ1* under conditions of salt stress demonstrated that* AcPsbQ1* may be involved in the response to salt stress in* A. canescens* [47].* Lhcb4.3* is unique among the photosystem II antenna proteins and is a determinant for photosystem II macroorganization and photoprotection [48, 49].* SBPASE* has been shown to influence photosynthetic capacity and function as a metabolic interface in oxidative stress, carbon assimilation, and multiple aspects of growth and development in Arabidopsis [50, 51].* PORC*—known to be involved in oxidoreductase activity—has been shown to regulate oxidative stress in Arabidopsis and protect plants from oxidative stress [52]. Here, in the present study the function of* Cas* is show as an electron carrier, while rice* OsCas* can decrease membrane damage and inhibition of photosynthesis under drought stress and has been previously shown to be involved in stress response and signaling pathways [53, 54]. Here, the functional analysis of genes coexpressed with* At5g17170* showed that the majority of the coexpressed genes are involved in the functioning of the chloroplast, which indicates that rubredoxin proteins might work in conjunction with other proteins in the chloroplast to perform their functions.

At the whole plant level, the effects of stress are usually reflected a decline in photosynthesis and slow growth and are associated with changes in carbon and nitrogen metabolism [55–57]. Research indicates that the chloroplast is closely associated with abiotic stress responses in plants, where the stability of the photosynthetic system is necessary to maintain the ability to photosynthesize in the face of environmental stimuli [58–62]. In the present study, although rubredoxin in maize did not exhibit significant changes in expression with salt or drought treatment, the development of expression profiles in* Z. mays* may provide new insights in the functions of rubredoxin. From aforementioned results, it is possible to hypothesize that rubredoxin localized to the chloroplast could act in the chloroplast system to respond to abiotic stress. However, for a complete picture of the role of rubredoxins in plants, further analyses will be necessary to determine their precise roles in responding to environmental stresses, as well as the underlying molecular mechanisms of stress resistance, using gene knock-out and overexpression mutants.

## 5. Conclusion

In conclusion, this study presents a stratagem to analyze and characterize rubredoxin in plants using bioinformatics-based techniques. A phylogenetic analysis of 66 rubredoxin candidates indicates that rubredoxin proteins in higher plants may have evolved from green algae. In addition, this exploration of evolutionary relationships of rubredoxin suggests that this analysis might be an important theoretical basis on which we further build the knowledge base regarding rubredoxin in plants. Rubredoxin proteins from different species share a high degree of similarity in structure, indicating that these rubredoxins may have similar functions in plants. The analysis of rubredoxin expression in different organs and over different development stages—as well as under salt stress and drought stress in glycophytic plants and a halophytic model plant—indicate that rubredoxin may be involved in diverse developmental process and stress responses in plants. Finally, a coexpression network of rubredoxin in* A. thaliana* was constructed to explore the interaction and pathways in which rubredoxin may participate. We found that the majority of genes in the coexpressed gene list of* At5g17170* were involved in responses to at least one abiotic stress and therefore have reasons to deduce that rubredoxin may play a role in responses to environmental adversity. Taken together, these results suggested that not only may rubredoxin function in the regulation of leaf development and growth, but also it may be involved in the stress response to salt and drought. Our study revealed that rubredoxin, localized to the chloroplast, with a PDZ domain near N-terminus and a rubredoxin domain in the C-terminal region may function in conjunction with other proteins in response to abiotic stress in the chloroplast. In conclusion, while we focused on the general features and functions of rubredoxin in plants, which will provide important information to the current base of knowledge and a point of reference for further functional analyses, there is much that remains to be elucidated about the molecular mechanisms of rubredoxin in plants.

## Figures and Tables

**Table 1:** Detected motifs of 66 rubredoxin-like proteins in plant with MEME tool.

motif	Nsites^a^	Width^b^	E-value^c^	motif regular expression
1	65	49	3.1e-3001	NAA[KR]AGLK[SA]GDQVLYTSSFFGDELWPADKLGFTKTAIQ AKPDSVYFVVS
2	65	53	9.4e-3171	THICLDCG[YF]IY[TF]L[PQ]K[PS]F[DE]EQPDTY[VGA]CPQC [INR]APKKRFA[RK]YDVNTG[KR][AP]IGGGLPP
3	66	29	1.2e-1561	GA[ED]VDVK[RK]LPKRPAPPRFGRKLT[ED][AT]QKAR
4	62	29	6.6e-1462	K[TN]IEVEVDKPLGLTLGQK[PQS]GGGVVIT[AG]V[ED]
5	62	21	1.2e-877	VI[IV]GL[VL]AG[IL][GA][AG]VGALLVYGLQ

^a^: Nsites indicated how many sequences have the corresponding motif sites.

^b^: width indicated how many amino acids in the predicted motif.

^c^: E-value means the statistical significance of a motif, which is based on its log likelihood ratio, its width, and number of occurrences.

## Data Availability

The data used to support the findings of this study are included within the article.

## Supplementary Materials

Supplementary 1 S1 File. Relevant GEO datasets used in the study.

Supplementary 2 S2 File. The statics information of identified candidate rubredoxin in plants.

Supplementary 3 S3 File. The chemical-physical characteristics of candidate rubredoxin.

Supplementary 4 S4 File. The significant function annotations of DAVID.
