# Supplementary material for: Molecular Evolution and Functional Analysis of Rubredoxin-Like Proteins in Plants
Source: Biomed Res Int. 2019 Jul 2;2019:2932585. doi: 10.1155/2019/2932585 (PMC6634066; doi:10.1155/2019/2932585)
Supplement: Supplementary 2 — S2 File. The statics information of identified candidate rubredoxin in plants. [file 2932585.f2.docx]

| **protein_ids** | **accsion_num** | **gene** | **length(bp)** |
| --- | --- | --- | --- |
| ABK21407_Psitchensis | ABK21407 | - | - |
| AFW57238_Zmays | AFW57238 | GRMZM2G079759 | - |
| AFW57239_Zmays | AFW57239 | GRMZM2G079759 |  |
| NP_001183375_Zmays | NP_001183375 | LOC100501788 | 2825 |
| EMS68403_Turartu | EMS68403 |  |  |
| XP_002443912_Sbicolor | XP_002443912 | LOC8069644 | 2933 |
| XP_004972763_Sitalica | XP_004972763 | LOC101784081 | 1772 |
| PutRub_Ptenuiflora | PutRub | - | - |
| XP_008776363_Pdactylifera | XP_008776363 | LOC103696486 | 6493 |
| NP_001061060_Osativa | NP_001061060 | Os08g0162600 | 2333 |
| XP_006659158_Obrachyantha | XP_006659158 | LOC102699330 | 2263 |
| XP_009391043_Mmalaccensis | XP_009391043 | LOC103977293 | 4030 |
| BAJ94117_Hvulgare | BAJ94117 | - | - |
| XP_010940800_Eguineensis | XP_010940800 | LOC105059265 | 7641 |
| XP_003573446_Bdistachyon | XP_003573446 | BRADI_3g16187 | 1747 |
| XP_010234369_Bdistachyon | XP_010234369 | LOC100831452 | 1747 |
| XP_002948168_Vocar | XP_002948168 |  | 2601 |
| XP_003080003_Otauri | XP_003080003 | OT_ostta06g03680 | 693 |
| XP_007513116_Bprasinos | XP_007513116 | Bathy05g04060 | 615 |
| XP_007029491_Tcacao | XP_007029491 | - | - |
| XP_010549812_Thassleriana | XP_010549812 | LOC104820874 | 2721 |
| XP_006347557_Stuberosum | XP_006347557 | LOC102602042 | 3138 |
| XP_004235309_Slycopersicum | XP_004235309 | LOC101255672 | 2762 |
| XP_011075820_Sindicum | XP_011075820 | LOC105160233 | 2205 |
| SaRub_Smongolica | SaRub | - | - |
| XP_002520001_Rcommunis | XP_002520001 | LOC8283059 | 1881 |
| XP_009361210_Pbretschneideri | XP_00936121 | LOC103951529 | 2122 |
| XP_007218777_Ppersica | XP_00721877 | LOC18785176 | 1728 |
| XP_008234595_Pmume | XP_008234595 | LOC103333524 | 1678 |
| XP_002325879_Ptrichocarpa | XP_002325879 | POPTR_0019s05950g | 2932 |
| XP_009621636_Ntomentosiformis | XP_009621636 | LOC104113228 | 4786 |
| XP_009782148_Nsylvestris | XP_009782148 | LOC104230935 | 3993 |
| XP_010255717_Nnucifera | XP_010255717 | LOC104596332 | 4059 |
| ACJ85407_Mtruncatula | ACJ85407 | - | - |
| XP_003610879_Mtruncatula | XP_003610879 | MTR_5g008050 | 2653 |
| XP_008376881_Mdomestica | XP_008376881 | LOC103439994 | 2052 |
| AFK49465_Ljaponicus | AFK49465 | - | - |
| XP_012090944_Jcurcas | XP_012090944 | LOC105649030 | 1825 |
| KJB18332_Graimondii | KJB18332 |  | - |
| KHN25601_Gsoja | KHN25601 | - | - |
| ACU19298_Gmax | ACU19298 | - | - |
| XP_003517534_Gmax | XP_003517534 | LOC100785369 | 2646 |
| XP_003537918_Gmax | XP_003537918 | LOC100816088 | 2494 |
| XP_004307912_Fvesca | XP_004307912 | LOC101312201 | 1705 |
| XP_006400247_Esalsugineum | XP_006400247 |  | 1947 |
| XP_010034959_Egrandis | XP_010034959 | LOC104424288 | 3009 |
| EYU45365_Mguttatus | EYU45365 | LOC105963982 | 1753 |
| XP_004143657_Csativus | XP_004143657 | LOC101215685 | 4112 |
| XP_008467304_Cmelo | XP_008467304 | LOC103504684 | 5220 |
| CDP04950_Ccanephora | CDP04950 | CDP04950 |  |
| XP_006491817_Csinensis | XP_006491817 | LOC102607698 | 2679 |
| XP_006428498_Cclementina | XP_006428498 | CICLE_v10012505mg | 2780 |
| XP_004511463_Carietinum | XP_004511463 | LOC101488830 | 2921 |
| XP_006288445_Crubella | XP_006288445 | - | 2067 |
| XP_010453899_Csativa | XP_010453899 | LOC104735749 | 2095 |
| XP_010492656_Csativa | XP_010492656 | LOC104770008 | 1921 |
| XP_009122863_Brapa | XP_009122863 | LOC103847521 | 1037 |
| XP_009126163_Brapa | XP_009126163 | LOC103851083 | 1654 |
| CDX85473_Bnapus | CDX85473 | BnaA02g03400D | - |
| CDY02732_Bnapus | CDY02732 | BnaC02g07030D | - |
| CDY16955_Bnapus | CDY16955 | BnaC09g40820D | 915 |
| XP_010671163_Bvulgaris | XP_010671163 | LOC104888040 | 3705 |
| KFK44649_Aalpina | KFK44649 | - |  |
| NP_568342.1_Athaliana | NP_568342 | ENH1 | 2149 |
| XP_002871751_Alyrata | XP_002871751 | - | 1,940 |
| XP_006854166_Atrichopoda | XP_006854166 | LOC18443924 | 4202 |

| **protein_ids** | | **Exon_num** | | **Chromo_loction** | **organism** |
| --- | --- | --- | --- | --- | --- |
| ABK21407_Psitchensis | - | | - | | Picea sitchensis |
| AFW57238_Zmays | 6 | | NC_024468.2(75764374 ..75767198, complement) | | Zea mays |
| AFW57239_Zmays | 6 | | NC_024468.2(75764374..75767462, complement) | | Zea mays |
| NP_001183375_Zmays | 6 | | NC_024468.2(75764374..75767198, complement) | | Zea mays |
| EMS68403_Turartu | 6 | | KD007000.1(145743..147171,complement) | | Triticum urartu |
| XP_002443912_Sbicolor | 6 | | NC_012876.2(5357780..5360712) | | Sorghum bicolor |
| XP_004972763_Sitalica | 6 | | NC_028455.1(3954556..3956327, complement) | | Setaria italica |
| PutRub_Ptenuiflora | - | | - | | Puccinellia tenuiflora |
| XP_008776363_Pdactylifera | 6 | | NW_008247502.1(24124..30616) | | Phoenix dactylifera |
| NP_001061060_Osativa | 6 | | NC_029263.1(3694274..3696648) | | Oryza sativa |
| XP_006659158_Obrachyantha | 7 | | NC_023170.1(2706447..2708709) | | Oryza brachyantha |
| XP_009391043_Mmalaccensis | 7 | | NC_025204.1(322848..326877) | | Musa acuminata subsp. malaccensis |
| BAJ94117_Hvulgare | - | | - | | Hordeum vulgare |
| XP_010940800_Eguineensis | 7 | | Chr16(6964059..6971672) | | Elaeis guineensis |
| XP_003573446_Bdistachyon | 6 | | NC_016133.2(14334951..14336697, complement) | | Brachypodium distachyon |
| XP_010234369_Bdistachyon | 6 | | NC_016133.2(14334951..14336697, complement) | | Brachypodium distachyon |
| XP_002948168_Vocar | 6 | | NW_003307552.1(729333..731933) | | Volvox carteri f. nagariensis |
| XP_003080003_Otauri | 1 | | NC_014431.2(631583..632275) | | Ostreococcus tauri |
| XP_007513116_Bprasinos | 1 | | NC_024004.1(782703..783317) | | Bathycoccus prasinos |
| XP_007029491_Tcacao | - | | - | | Theobroma cacao |
| XP_010549812_Thassleriana | 7 | | NW_010966372.1:574152..576872 | | Tarenaya hassleriana |
| XP_006347557_Stuberosum | 6 | | NW_006239018.1(743461..746598) | | Solanum tuberosum |
| XP_004235309_Slycopersicum | 6 | | Chr3:(59230653..59233414) | | Solanum lycopersicum |
| XP_011075820_Sindicum | 6 | | NC_026148.1(8379870..8382074, complement) | | Sesamum indicum |
| SaRub_Smongolica | - | | - | | Salix mongolica |
| XP_002520001_Rcommunis | 6 | | NW_002994350.1:234552..236432 | | Ricinus communis |
| XP_009361210_Pbretschneideri | 6 | | NW_008988121.1(338920..341041, complement) | | Pyrus x bretschneideri |
| XP_007218777_Ppersica | 6 | | NC_034010.1(29964884..29966611) | | Prunus persica |
| XP_008234595_Pmume | 6 | | NC_024130.1(24933913..24935590, complement) | | Prunus mume |
| XP_002325879_Ptrichocarpa | 6 | | LGXIX:6365375..6368306 | | Populus trichocarpa |
| XP_009621636_Ntomentosiformis | 6 | | NW_008905907.1(13612..18397) | | Nicotiana tomentosiformis |
| XP_009782148_Nsylvestris | 6 | | NW_009390048.1(27246..31238) | | Nicotiana sylvestris |
| XP_010255717_Nnucifera | 6 | | NW_010729089.1:643424..647519 | | Nelumbo nucifera |
| ACJ85407_Mtruncatula | - | | - | | Medicago truncatula |
| XP_003610879_Mtruncatula | 5 | | NC_016411.2(1646673..1649325) | | Medicago truncatula |
| XP_008376881_Mdomestica | 6 | | NC_024245.1:25960175..25962226 | | Malus domestica |
| AFK49465_Ljaponicus | - | | - | | Lotus japonicus |
| XP_012090944_Jcurcas | 6 | | NW_012125415.1(268590..270414, complement) | | Jatropha curcas |
| KJB18332_Graimondii | 6 | | - | | Gossypium raimondii |
| KHN25601_Gsoja | 6 | | - | | Glycine soja |
| ACU19298_Gmax | - | | - | | Glycine max |
| XP_003517534_Gmax | 6 | | Chr1(55744682..55747327) | | Glycine max |
| XP_003537918_Gmax | 6 | | Chr11(887038..889531) | | Glycine max |
| XP_004307912_Fvesca | 6 | | LG7(20493922..20495626) | | Fragaria vesca |
| XP_006400247_Esalsugineum | 6 | | NW_006256829.1(5776009..5777955) | | Eutrema salsugineum |
| XP_010034959_Egrandis | 6 | | NW_010092448.1(1622355..1625363) | | Eucalyptus grandis |
| EYU45365_Mguttatus | 6 | | NW_012192848.1(697880..699551) | | Erythranthe guttata |
| XP_004143657_Csativus | 6 | | NC_026659.1(6702419..6706530) | | Cucumis sativus |
| XP_008467304_Cmelo | 6 | | NW_007546272.1(5067523..5072742) | | Cucumis melo |
| CDP04950_Ccanephora | 6 | | - | | Coffea canephora |
| XP_006491817_Csinensis | 6 | | NW_006257078.1(1496540..1499218) | | Citrus sinensis |
| XP_006428498_Cclementina | 6 | | NW_006262139.1(5908592..5911371) | | Citrus clementina |
| XP_004511463_Carietinum | 6 | | NC_021167.1(1084732..1087652) | | Cicer arietinum |
| XP_006288445_Crubella | 6 | | NW_006238918.1(672970..5675036) | | Capsella rubella |
| XP_010453899_Csativa | 6 | | Chr13(7351914..7354008) | | Camelina sativa |
| XP_010492656_Csativa | 6 | | Chr20(8267297..8269217) | | Camelina sativa |
| XP_009122863_Brapa | 5 | | A10(12887139..12888175) | | Brassica rapa |
| XP_009126163_Brapa | 6 | | A2(4138083..4139736) | | Brassica rapa |
| CDX85473_Bnapus | 6 | | - | | Brassica napus |
| CDY02732_Bnapus | 6 | | - | | Brassica napus |
| CDY16955_Bnapus | 7 | | LK032068(562739-564236) | | Brassica napus |
| XP_010671163_Bvulgaris | 6 | | NC_025814.2(2273881..2277585, complement) | | Beta vulgaris |
| KFK44649_Aalpina | 6 | | - | | Arabis alpina |
| NP_568342.1_Athaliana | 6 | | Chr5(5649057..5651205) | | Arabidopsis thaliana |
| XP_002871751_Alyrata | 6 | | NW_003302550.1(7047655..7049777) | | Arabidopsis lyrata |
| XP_006854166_Atrichopoda | 6 | | NW_006499913.1(3426278..3430479) | | Amborella trichopoda |
